# Supplementary material for: The Activity of Human NK Cells Towards 3D Heterotypic Cellular Tumor Model of Breast Cancer
Source: Cells. 2025 Jul 8;14(14):1039. doi: 10.3390/cells14141039 (PMC12293796; doi:10.3390/cells14141039)
Supplement: Supplementary file 1 [file cells-14-01039-s001.zip › cells-3718302-supplementary.pdf]

Supplementary Materials

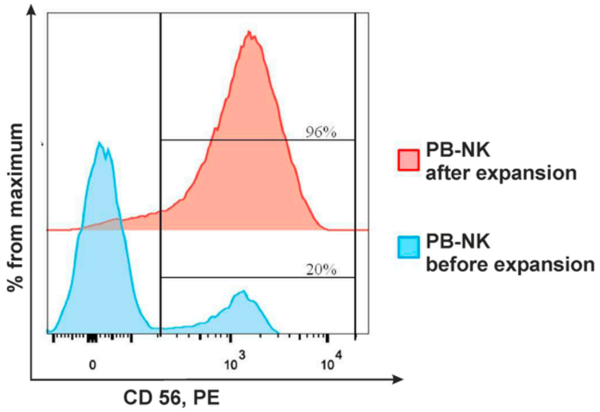

Figure S1. Isolation and activation of human NK cells from healthy donors using RosetteSep Human NK Cell Enrichment Cocktail. IL-15 and IL-2 are added to the resulting immune cells when PB-NKs are isolated from peripheral blood (PB) to maintain their viability.

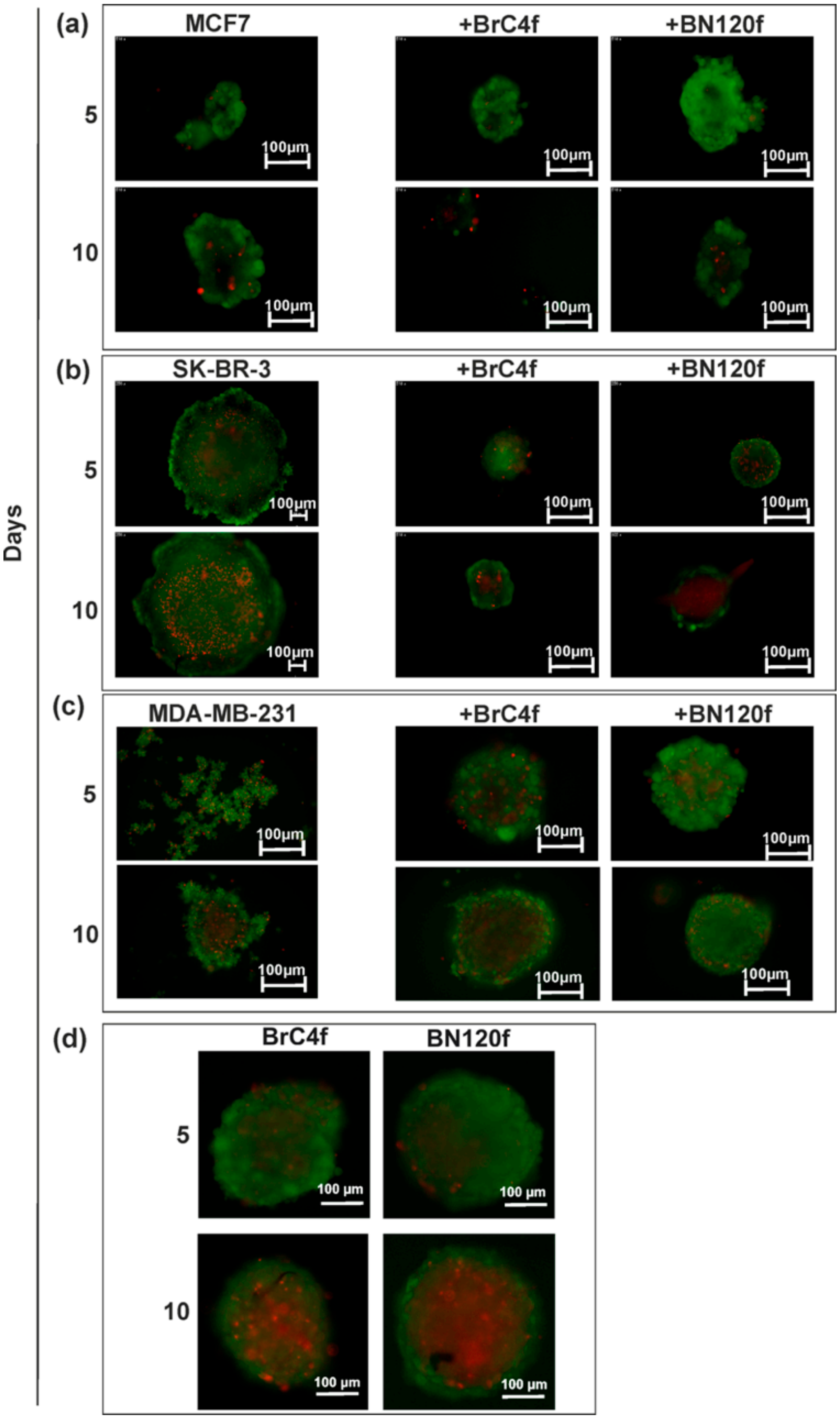

Figure S2. Images of Live/Dead-stained homo- and heterotypic spheroids (**a**) MCF7; (**b**) SK-BR-3; (**c**) MDA-MB-231; (**d**) stromal cells. The cells in spheroids were stained with FDA (green, live cells) and PI (red, dead cells) on Day 5 and Day 10.

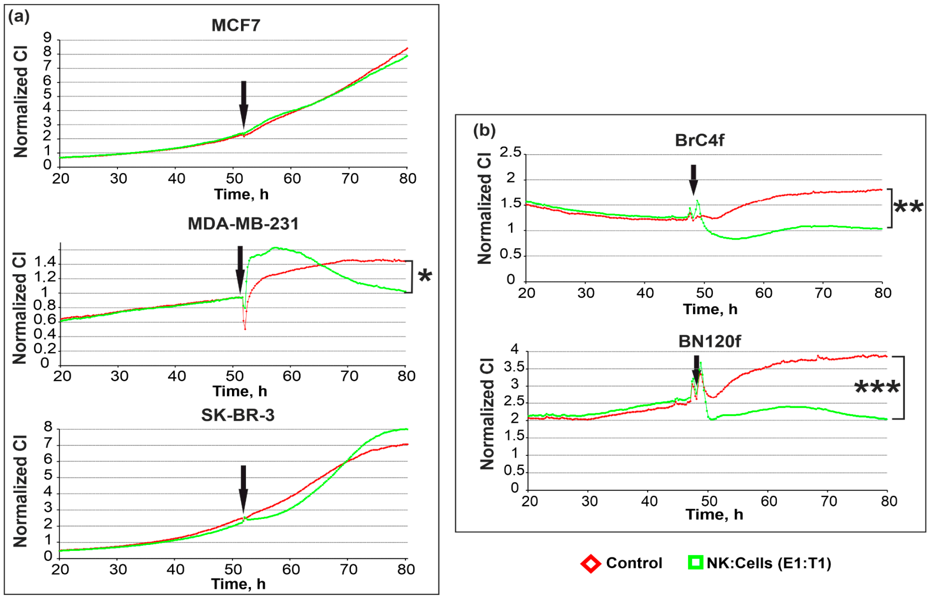

Figure S3. Killer activity of NK cell line YT against tumor (**a**) and stromal (**b**) cells in 2D model. Target cells were seeded with a density of 30,000 cells/well. After 52 h NK-YT cells were added at 1:1 E:T ratio. Impedance was measured at well-bottoms every 15 min for 80 h. Changes in impedance were given as dimensionless cell index (CI). Target cells alone served as control. * $p < 0.05$; ** $p < 0.01$; *** $p < 0.001$ determined by unpaired two-tailed Student's t test.

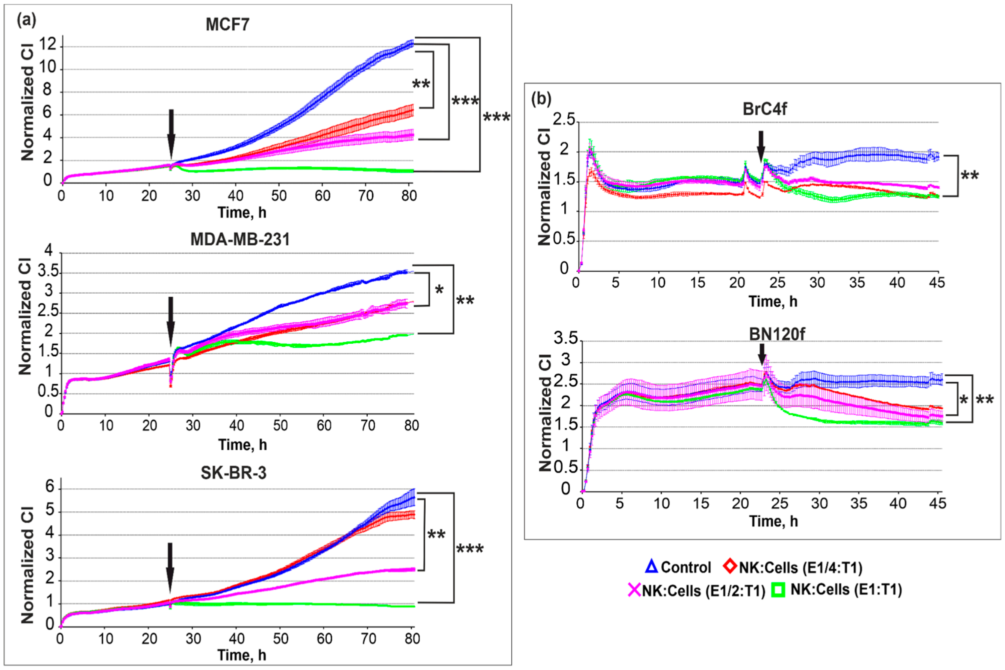

Figure S4. Analysis of killer activity of PB-NK cells on tumor (**a**) and stromal (**b**) cells by xCELLigence real-time cell analyzer. Target cells were grown in multiple wells of an E-Plate. Different quantities of PB-NK cells were added to each well and impedance was monitored continuously for the next 50 hours. * $p < 0.05$; ** $p < 0.01$; *** $p < 0.001$ determined by unpaired two-tailed Student's t test.The addition of PB-NK cells is indicated by an arrow.

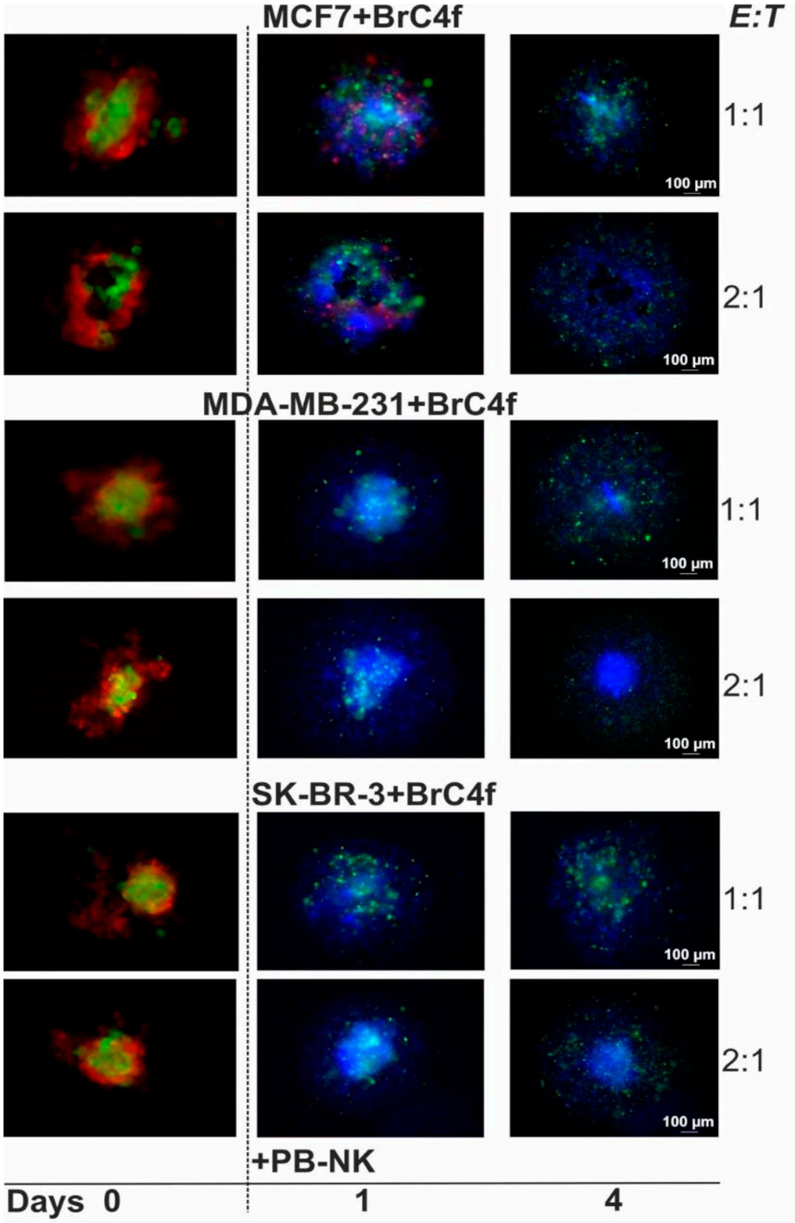

Figure S5. Cytotoxic effect of PB-NK cells in heterotypic spheroid at high E:T ratio. Fluorescent images of tumor (red), CAFs (green) and NK (blue) cells in E1:T1 or E2:T1 ratio on 1 and 4 days of co-culture.

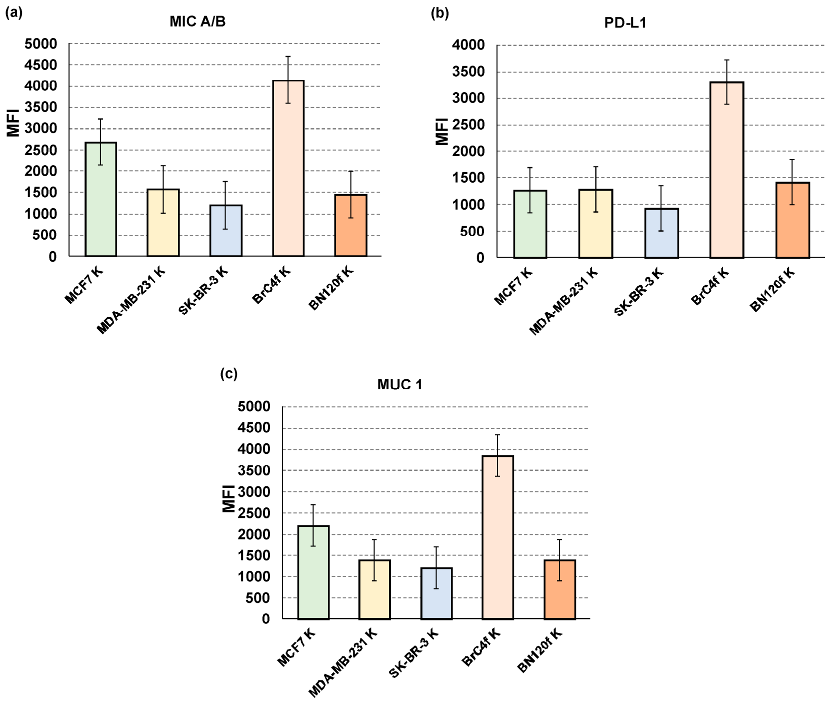

Figure S6. MFI values of (**a**) MICA/B, (**b**) PD-L1 and (**c**) MUC1 in cells of 2D. MFI - mean fluorescent intensities. * $p < 0.05$; ** $p < 0.01$; *** $p < 0.001$ determined by unpaired two-tailed Student's t test. Flow cytometry analysis.

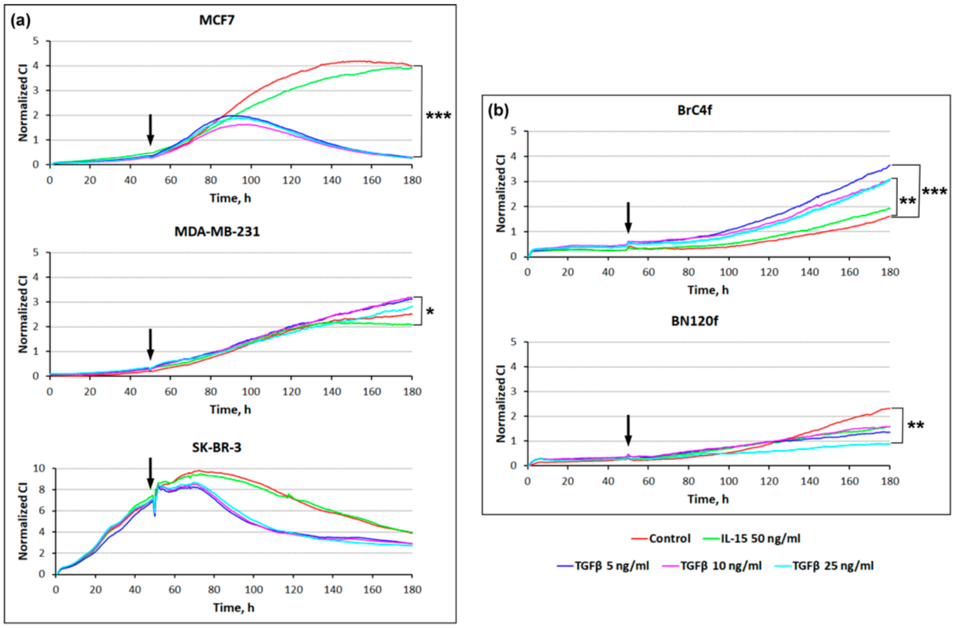

Figure S7. The effect of combination cytokines IL-2 (300 units/ml) with IL-15 (50 ng/ml) or TGFβ (5, 10, 25 ng/ml) on the viability and proliferation of (**a**) tumor and (**b**) stromal cells. The cell growth curves in real time are presented, with the moment of cytokine addition indicated by the arrow. The Cell Index (CI) is the cell proliferation index (relative units). * $p < 0.05$; ** $p < 0.01$; *** $p < 0.001$ determined by unpaired two-tailed Student's t test.

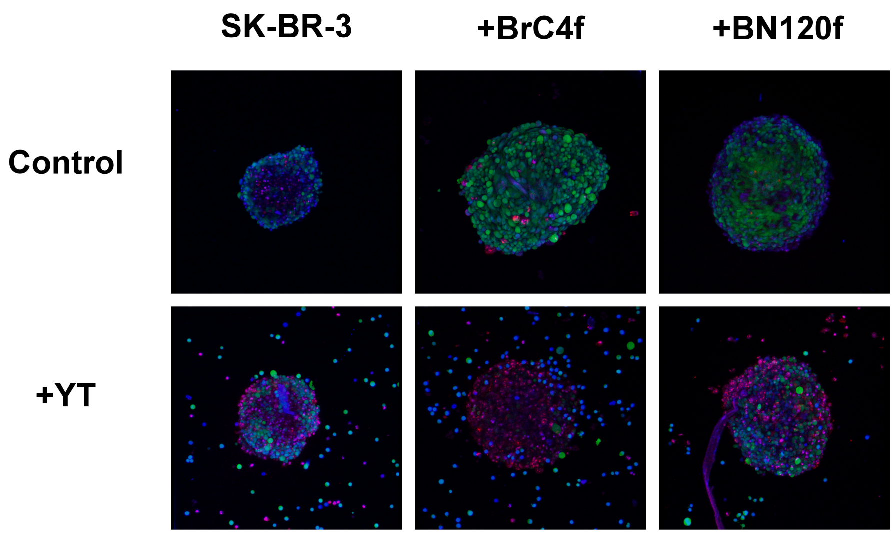

Figure S8. Cell viability in 3De and 3D-2 spheroids from SK-BR-3 on 7 days of total cultivation and 3 days with NK-cells. The green signal indicates viable cells, the red signal indicates dead cells, and the blue signal indicates the total number of cells. The samples were observed using confocal microscopy with a magnification of 10x.

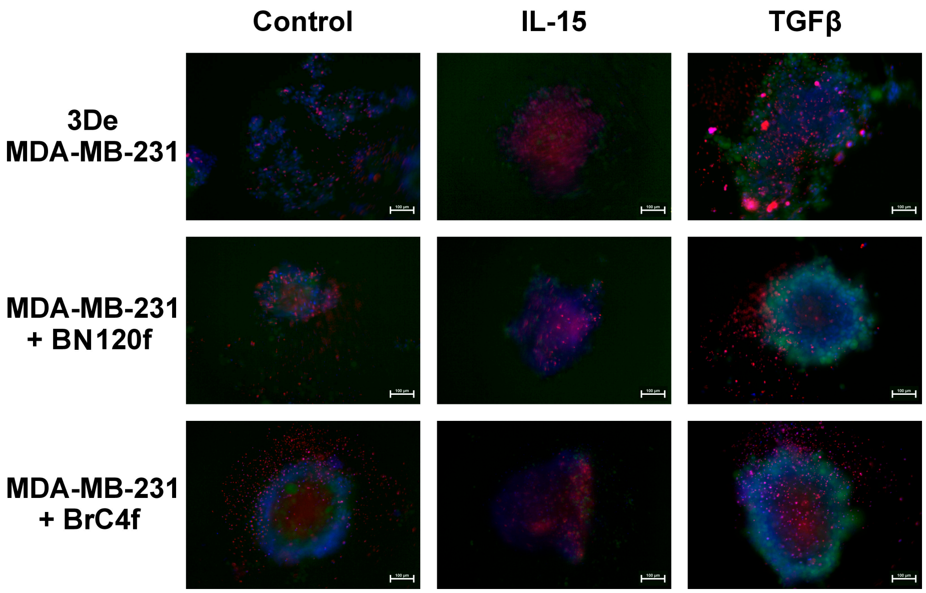

Figure S9. Representative images the TGFβ (5 ng/ml) inhibitor activity of PB-NK cells in heterotypic 3D-2 models from MDA-MB-231 is clearly evident, as is the increase in proliferation of the MDA-MB-231 cells. Green signal (FDA) - live cells, red (PI) - dead cells, blue (Hoechst 33342)- total number of cells. Monitoring of cells were observed during a 5-day co-culture period.

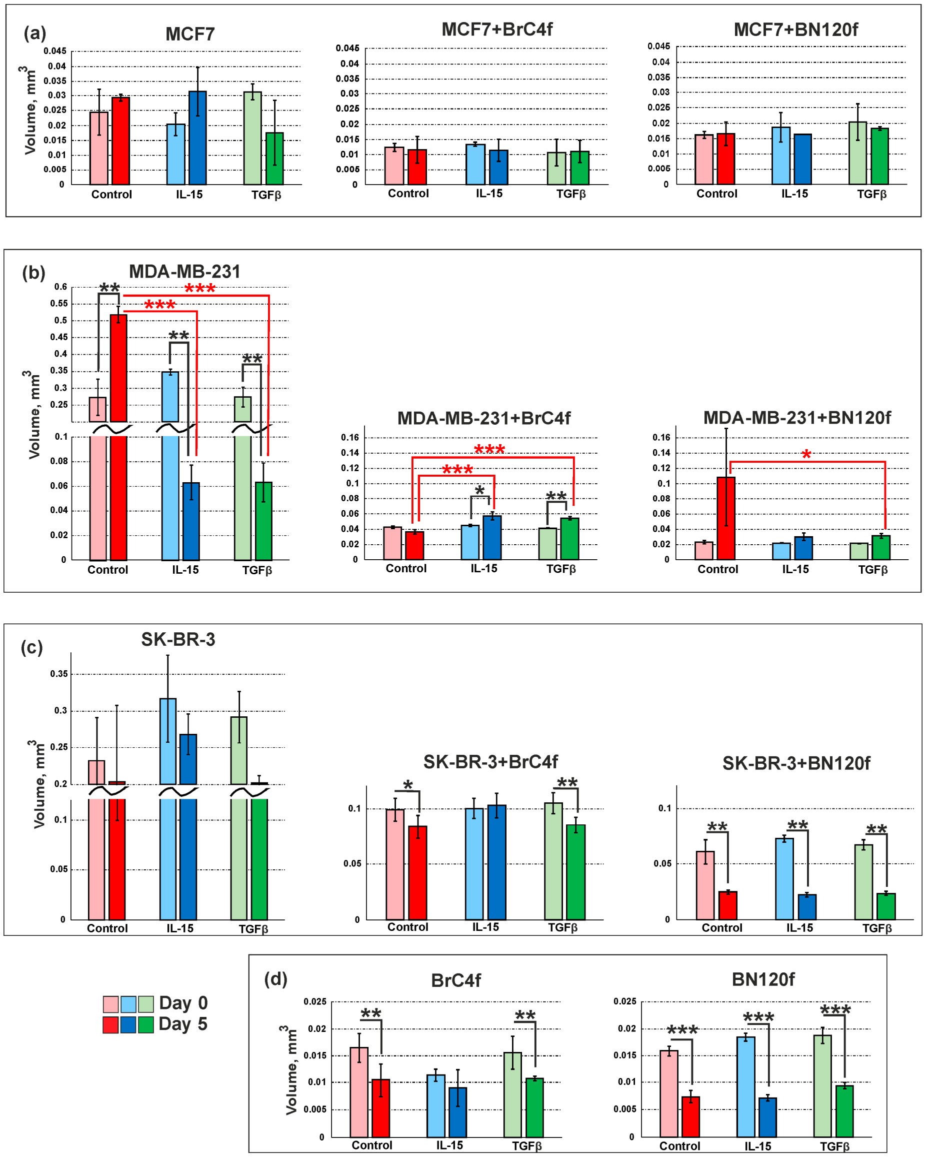

Figure S10. Volume of homo- and heterotypic spheroids (**a**) MCF7; (**b**) MDA-MB-231; (**c**) SK-BR-3; (**d**) stromal cells after co-cultivation with PB-NK in the presence combination IL-2 (300 units/ml) with IL-15 (50 ng/ml) or TGFβ (5 ng/ml). Data presented as mean ± SD, * $p < 0.05$; ** $p < 0.01$; *** $p < 0.001$, using ANOVA.
